# Concept of Lethal Triad in Critical Care of Severe Burn Injury

**DOI:** 10.5005/jp-journals-10071-23161

**Published:** 2019-05

**Authors:** Vamseedharan Muthukumar, Durga Karki, Bhojani Jatin

**Affiliations:** 1-3 Department of Burns, Plastic and Maxillofacial Surgery, VM Medical College and Safdarjung Hospital, New Delhi, India

**Keywords:** Burns, Critical care, Lethal triad, Mortality

## Abstract

**Background:**

The trinity of hypothermia, acidosis and coagulopathy, the lethal triad in trauma setting is a well-known risk factor associated with high risk of death. Burn is also a pathological situation where inflammatory response, endothelial injury, hypovolemia, reduced end-organ perfusion, cellular hypoxia, and myocardial depression are frequently encountered. This study aimed to study the occurrence and outcome of patients presenting with the ‘triad of death’ in burn population.

**Methods:**

The study population included patients between 18 years and 60 years presenting to the department with thermal and scald burns with total body surface area involving 50–70%. The study was conducted for a period of 180 days and patients were followed up for 30 days. A *p* value <0.05 was considered statistically significant.

**Results:**

One hundred and ninety-six patients were admitted during study period. Fifty patients out of them were eligible and were included in the study. The average abbreviated burns score index was 11 in lethal triad subgroup when compared to eight in the subgroup without the lethal triad. The mortality in the subgroup with lethal triad was higher (68.8% vs 17.6%, *p* = 0.0009). The “on admission” acidosis, hypothermia, and coagulopathy were independently associated with significantly increased mortality. The overall relative risk of mortality in the presence of lethal triad was 3.896.

**Conclusion:**

This study reiterates the fact that the lethal triad is seen in burn patient. Burn associated with on admission lethal triad has significantly higher mortality rates. There are only countable studies addressing this issue in burn setting.

**How to cite this article:**

Muthukumar V, Karki D, Jatin B. Concept of Lethal Triad in Critical Care of Severe Burn Injury. Indian J Crit Care Med 2019;23(5):206-209.

## BACKGROUND

The trinity of hypothermia, acidosis and coagulopathy, known as the lethal triad, in trauma setting is a well-known risk factor associated with high risk of death.^1^ The occurrence of this deadly triad not only depends on the severity of injury, but also relates to the resuscitative interventions received during the course of the management.^2^ In trauma setting, the concept of damage control resuscitation has taken the forefront with fruitful results. The damage control measure entails damage control surgery, hemostatic resuscitation and permissive hypotension, and has been associated with improved survival in trauma patients with severe hypervolemia.^3^ These three can develop quickly in the hypovolemic trauma patient and once sets in, it forms a vicious circle that may be difficult to surmount.

Burn, in actual terms, is also a trauma setting where these three factors are encountered. Burn is also a pathological situation where inflammatory response, endothelial injury, hypovolemia, reduced end-organ perfusion, cellular hypoxia, and myocardial depression are frequently encountered. In burn, isolated occurrence of acidosis, hyperlactemia, coagulopathy, and hypothermia are known entities associated with raised mortality rates. However, there are not much of the data available in world literature regarding the occurrence of the triad in burn setting.^4^ Also, the phenomenon of damage control measures has not gained pace in burn injury and most of the centers still continue to follow the resuscitation according to the preformed formulae and protocols.

This study aimed to analyze the occurrence and outcome of patients presenting with the ‘triad of death’ in burn population. This was also undertaken to outline any change in the outcome of such patients over time.

## METHODS

### Study Setting

The study was conducted at a premiere tertiary care center for burns in India.

### Study Population

The study population included patients between 18 years and 60 years presenting to the department with thermal and scald burns with total body surface area involving 50–70%. The study was conducted for a period of 180 days and patients were followed up for 30 days. The exclusion criteria included associated trauma other than burns, presentation to our center after 24 hours from time of burn, preexisting comorbidities including bleeding diathesis and drugs causing impairment of coagulation profile, use of blood products, and missing fluid data in referral cases.

### Data and Definitions

Data for all eligible patients were collected at the time of admission and were recorded in a preformed template using Microsoft Excel 2010 (Microsoft, USA) with anonymity. From the available literature, the following definitions were arrived at:

- Hypothermia was defined as a temperature of less than or equal to 35.5 °C.
- Acidosis was defined as pH of less than or equal to 7.25.
- Coagulopathy was defined as an International normalized ratio (INR) greater than 1.2.

The patients satisfying the three criteria at the time of admission were considered to possess the deadly triad.

### Statistical Analysis

The population was divided into patients with and without the deadly triad. The incidence of the triad was calculated. Data were analyzed for any association between the deadly triad with mortality. The skewed data were represented as mean and median. The continuous variables were analyzed with t test. A *p* value <0.05 was considered statistically significant.

## RESULTS

One hundred and ninety-six patients were admitted during the 180-day time period. One hundred and forty-six patients did not meet the eligibility criteria and were excluded from this study. Fifty patients out of them were eligible and were included in the study (Flowchart 1). The mean age and weight of included population were 42.3 years and 62.5 kg, respectively. There was a male preponderance seen in the included population. The mean total burn surface area (TBSA) was 56.5, and inhalational and full thickness burns were seen in 21 and 19 patients, respectively (Table 1). The lethal triad subgroup had 16 patients. This sub group had mean age of 51.3 years and mean weight of 67.2 kg. The TBSA was significantly higher and the incidence of inhalational injury and full thickness burns were significantly higher (14 and 10 patients, respectively) when compared to the subgroup without the lethal triad. The average abbreviated burns score index (ABSI) was 11 in lethal triad subgroup when compared to eight in the subgroup without the lethal triad. Though the total fluids received prior to the arrival and the fluid deficit at the time of arrival was not statistically significant. The mean fluid deficit was much higher in the group with lethal triad when calculated according to the modified Brookes formula (first 24 hours: Ringer lactate at 2 mL/kg/% TBSA burn, one half in the first 8 hours and half in the remaining 16 hours; and second 24 hours: half of the fluid calculated in the first 24 hours + maintenance fluid to maintain urine output).

**Flowchart 1:**
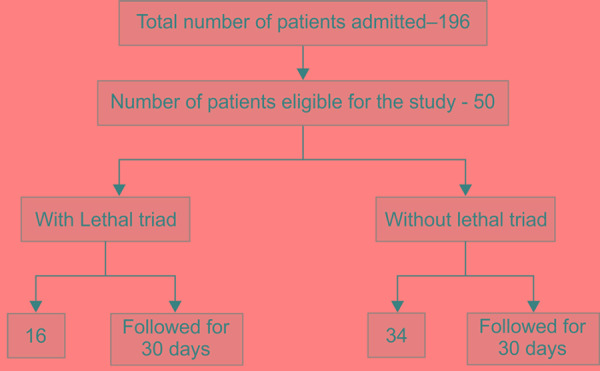
Flow of study population

**Fig. 1:**
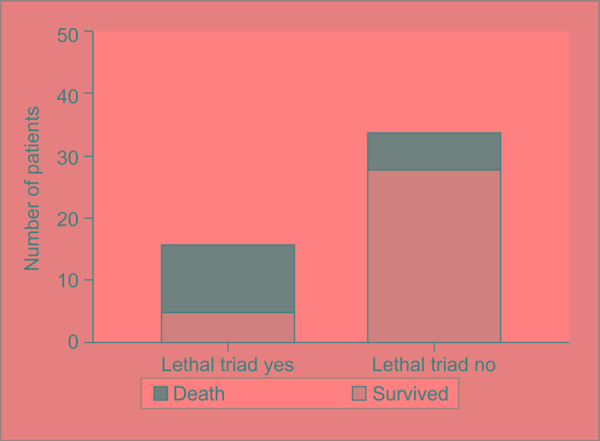
Comparison of mortality in the subgroups

**Fig. 2:**
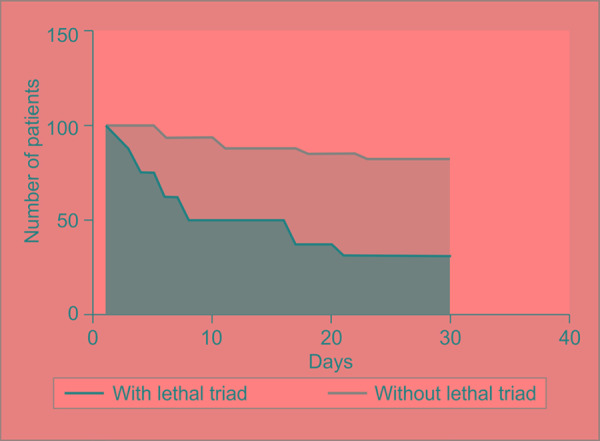
Trend of survival in the subgroups

The mortality in the subgroup with lethal triad was higher (68.8% vs 17.6%, *p* = 0.0009) (Fig. 1). The individual components were studied for associated mortality (Table 2). The “on admission” acidosis, hypothermia, and coagulopathy were independently associated with significantly increased mortality. The overall relative risk of mortality in the presence of the lethal triad was 3.896. The trend of the patient survival in the both groups was plotted against the duration of study in days (Fig. 2). The trend analysis showed significantly reduced survival with 50% mortality as early as eight days in the subgroup with lethal triad.

## DISCUSSION

Since the understanding of the deadly triad started in early 1990s, the triad of hypothermia, hypercoagulability, and acidosis have been extensively studied in trauma situations.^1^ This complex triad with multiple aspects make the condition difficult to reverse or control. The adverse effects of the individual constituent of the triad have formerly been described and associated with poor prognosis in trauma patients.^5^ Hypothermia contributes to mortality over and above the mortality associated with multiple severe injuries, independent of hypotension, fluid requirements, age, or duration of surgery. When patients are matched for injury severity, hypothermia has been shown to increase the mortality.^6^ Hypothermia is a significant contributor to coagulopathy and independent of acidosis or the amount of fluid infused.^7^ Coagulopathy on presentation has been associated with a four to five fold increase in overall mortality and early death post-major trauma.^8^ Metabolic acidosis in major trauma patients has also been independently associated with mortality with severity predictive of the outcome in critically ill patients.^9^ The concurrent presence of all three conditions not only adds to mortality, but further potentiates other constituents of the triad, forming what has been described as a “vicious cycle resulting in death”.

**Table 1:** Demographics and characteristics of study population

*Demographics*	*Overall* *n = 50*	*Lethal triad*		*p value*
		*Yes (n = 16)*	*No (n = 34)*	
Age in years, mean	42.3	51.3	38.1	0.0001
Weight in kg, mean	62.5	67.2	60.3	0.1904
Gender (M/F)	28/22	10/6	18/16	
TBSA burn, mean	56.5	66.2	51.9	<0.0001
Inhalational injury present	21	14	7	<0.0001
Full-thickness burn present	19	10	9	0.0038
ABSI, mean	9	11	8	<0.0001
Time from burn to arrival (minutes), mean	428.5	543.1	374.6	0.0187
Fluid received prior to arrival (ml), mean	3200	4100	2800	0.0473
Fluid deficit according to modified brooke's on arrival (ml), mean	–1800	–2300	–1550	0.0519
INR, mean	1	1.5	0.7	<0.0001
Temperature in celsius, mean	35.9	34.8	36.4	<0.0001
pH, mean	7.29	7.15	7.36	<0.0001
Death within 30 days (%)	17/50 (34)	11/16 (68.8)	6/34 (17.6)	0.0009

**Table 2:** Relation of individual parameters of lethal triad on mortality

*Category*	*Present*	*N*	*Death within 30 days*	*p value*	*Katz-Koopman asymptotic risk score*	*Relative risk*
Coagulopathy	Yes	20	13	0.0002	0.4038	4.875
	No	30	4			
Acidosis	Yes	21	15	<0.0001	0.3069	10.36
	No	29	2			
Hypothermia	Yes	18	12	0.0005	0.3951	4.27
	No	32	5			
Lethal triad	Yes	16	11	0.0009	0.3795	3.896
	No	34	6			

Burns is also a trauma setting in which physiology is altered and is pathologically associated with hypotension, hypoperfusion, and anaerobic metabolism. The reserves of the patient are so much utilized in the acute phase, so as even subtle fluctuations in patient condition tilt the prognosis balance adversely.^10^ Impaired skin's insulating ability and reduced capability of endogenous heat production contributes to hypothermia in the burns patients’ especially elderly subgroup. Anaerobic metabolism restricts the stock of adenosine triphosphate, which is essential for the endogenous heat production.^11^ Despite these factors, the occurrence of hypothermia in burn patients is trifling. The blend of impaired endogenous heat production, reduced basal metabolic rate and nutrition, especially in the elderly age, makes them prone to the lethal triad.

The severe the burn, more it is related to wide spread endothelial dysfunction, acute phase response and volume depletion, resulting in compromised myocardial contractility and end organ perfusion. The impaired end organ perfusion results in anaerobic cellular metabolism, increased lactic acid, and acidosis.^12^ The falling base reserves, raising the base deficit, and lactic acid levels are significant biomarkers of end organ hypoperfusion, and are robust markers associated with high morbidity and mortality in burn patients.^13^ Impaired tissue perfusion might also be associated with coagulopathy resulting from for early endogenous anticoagulation and fibrinolysis. The initial in-house pH was found to be a good predictor of death in severe burn patients.^14^

The inadequate fluid resuscitation, high tissue injury burden and in combination with resulting endothelial disruption, and poor heat insulation may drive the lethal triad in severe burns.^13^ Burns patients presenting with lethal triad at admission have high mortality when compared than those without lethal triad.^4^

The fundamental to the management of the severe thermal injuries in early period pivots about the accurate assessment of body surface area involved and timely and adequate resuscitation to prevent burn shock.^13^ Accurate body surface area estimation combined with balanced fluid resuscitation would optimistically mitigate end organ hypoperfusion, acidosis, and burn shock. Active and passive warming strategies should be utilized early to allay heat losses during resuscitation. This plan of resuscitation combined with heat conservation strategies can possibly restore the battered physiology to milieu interior. With these measures, it may be conceivable to reduce the occurrence of the lethal triad.

## LIMITATIONS

This was a single centre study with limited population eligible for the study. Further studies with larger population can throw more light on the impact of this study.

## CONCLUSION

This study reiterates the fact that the lethal triad is seen in the burns patients. Lethal triad is more commonly associated with severe burn injury (higher TBSA burnt and associated inhalational burns and full thickness burns). Burns associated with on admission lethal triad has significantly higher mortality rates. There are only countable studies addressing this issue in burns setting.

## FUTURE DIRECTION

Arriving at this conclusion deciphers only the portion of the key glitch burn surgeons’ face. In future, it needs to be seen whether the identified lethal triad is a modifiable risk factor in severe thermal injuries with impact on mortality and whether the concept of damage control resuscitation is feasible in burns patients and its outcome in terms of morbidity and mortality. This would require further prospective trials on a multicentre level to arrive at consensus.
